# Annual alveolar bone loss in older adults taking oral bisphosphonate: a retrospective cohort study

**DOI:** 10.1186/s12903-019-0955-6

**Published:** 2019-11-27

**Authors:** Mohammad Helmi, Sara AlOsaimy, J. Max Goodson, Hatice Hasturk, Zuhair S. Natto

**Affiliations:** 1 Periodontics and Community Dentistry Department, College of Dentistry, King Saud University, Riyadh, Saudi Arabia; 2 Eugene Applebaum College of Pharmacy and Health Sciences, Detroit, Michigan USA; 3 College of Pharmacy, King Saud University, Riyadh, Saudi Arabia; 4 Department of Oral Medicine, Infection, and Immunity, School of Dental Medicine, Harvard University, Boston, MA USA; 5 Department of Applied Oral Sciences, Center for Clinical and Translational Research, The Forsyth Institute, Cambridge, MA USA; 6 Department of Dental Public Health, School of Dentistry, King Abdulaziz University, Jeddah, Saudi Arabia

**Keywords:** Bisphosphonate, Periodontal disease, Annual bone loss, Predict

## Abstract

**Background:**

Although several studies assessed the effect of bisphosphonate (BIS) administration on alveolar bone loss, this relationship has not been fully investigated using longitudinal analysis. The aim of the this article is to predict annual alveolar bone loss in a subpopulation of older adults patients who were taking oral bisphosphonate (BIS), adjusting for systemic diseases and associated risk factors.

**Methods:**

This is a retrospective cohort study. We identified all subjects who reported receiving oral bisphosphonate from 2008 to 2015 (*N* = 30) using the electronic health records of each patient to identify suitable radiographs for analysis. For the longitudinal data analysis, 26 subjects were eligible for inclusion, having at least two exposures of the complete mouth set or repeated bitewing radiographs at least a one-year interval; they were then matched on age and sex to another 26 patients who did not report receiving bisphosphonate at any point of their life.

**Results:**

Mild periodontitis was higher in the BIS group compared to the no BIS group; however, moderate periodontitis was higher in the no BIS group. For those who did not take oral BIS, change over time was not significant after the two-year period. However, the BIS group had experienced 0.088 mm more bone loss compared to the no BIS group (95% CI: 0.001, 0.176. *P*-value = 0.048), adjusting for all other variables included in the model.

**Conclusion:**

The group that reported receiving oral bisphosphonates showed no improvement in maintaining alveolar bone level, and the use of oral BIS may not be effective in reducing annual alveolar bone loss; however, emerging evidence is promising for the use of bisphosphonate as an adjunctive local delivery medication for the management of periodontal diseases.

## Background

Although several studies raised the question of whether increased alveolar bone loss is a natural consequence of aging [1–5], a higher prevalence of periodontitis and bone loss in general have been consistently addressed and reported in the literature to be associated with aging [4–9].

Periodontitis was also reported in the literature to be associated with age-related diseases, such as osteoporosis, especially in postmenopausal women [10–13]. In 2018, Mashalkar et al. published a study on postmenopausal women to investigate the correlation between periodontitis and osteoporosis [14]. Authors of the study concluded that there was significant association between osteoporosis in postmenopausal women and the severity of periodontitis.

Multiple studies also assessed the effect of bisphosphonate (BIS) administration on alveolar bone loss [15–17]. Bisphosphonates were introduced to clinical practice decades ago [18, 19]. They are structurally related to inorganic pyrophosphate, as they contain a core phosphate-carbon-phosphate structure with the highest affinity for the bone relative to other tissues. Bisphosphonates inhibit enzymatic degradation, hinder calcification and suppress bone resorption. They are utilized in conditions where there is an imbalance between osteoblast-mediated bone formation and osteoclast bone resorption.

Bisphosphonates are the mainstay of therapy for skeletal disorders, particularly osteoporosis due to skeletal remodeling, because they achieve a high concentration to active bone remodeling sites, with conditions such as those with accelerated skeletal turnover [18–20]. They increase the density of the bone, reduce markers of bone turnover and ultimately reduce fractures [19]. In addition, bisphosphonates are utilized to resolve hypercalcemia among cancer patients [18–20]. Other clinical implications include: primary hyperparathyroidism, osteogenesis imperfecta and paget’s disease of bone [20]. However, it may lead to bisphosphonate-related osteonecrosis of the jaw (BRONJ), which was renamed to medication-related osteonecrosis of the jaw (MRONJ). This is related other bone resorption inhibitors or angiogenesis inhibitors, due to the excessive inhibition of angiogenesis and jaw metabolic processes, toxicity, inflammation, immunity disorder and infection [21, 22].

Due to its marked efficacy in the prevention of bone loss in susceptible populations, alendronate (generic name of BIS) had been proposed as a useful agent to prevent alveolar bone loss [22]. One systematic review assessed eight clinical studies that evaluated the efficacy of bisphosphonate therapy in the management of periodontitis, particularly as an adjunct to scaling and root planing [23]. Alendronate was utilized as either a topical application or oral therapy option. The study concluded that there was a statistically significant reduction in probing depth and bone defect, suggesting the clinical effectiveness of bisphosphonate in the management of periodontitis.

Another group investigated the potential outcomes of alendronate among postmenopausal women with periodontal disease [24]. Postmenopausal women are at the highest risk for osteoporosis due to estrogen deficiency. Authors of the study concluded that oral alendronate improved periodontal health and alveolar bone turnover in postmenopausal women.

Moreover, El-Shinnawi et al. in 2003 published a clinical trial on 24 adults with periodontitis that had been followed for 6 months [25]. Twelve patients were administered oral alendronate and were compared to a control group that did not receive any drug. Although clinical parameters (attachment level, pocket depth and gingival index) of the alendronate group showed no difference compared to the control group, the alendronate group showed a significant change in bone density compared to the control group, favoring patients who received oral bisphosphonate. For this reason, the aim of this study is to evaluate annual alveolar bone loss in a subpopulation of older adults patients who were taking oral bisphosphonate, adjusting for systemic diseases and associated risk factors. We hypothesized that BIS patients would have less alveolar bone loss compared with no BIS.

## Methods

This is a retrospective cohort study that followed STROBE checklist. It was approved by the office of human research administration, Harvard Faculty of Medicine, [45 CFR 46.101(b) (4)], #IRB 16–1838. We collected records of all patients that reported receiving oral BIS from 2008 to 2015 (*N* = 30), to identify suitable radiographs for analysis. To be included in the study, each patient should have at least two exposures of Complete Mouth Radiographic Series (CMRS) or repeated bitewing (BW) radiographs at least a one-year interval. Furthermore, each BW radiograph had to clearly show the alveolar bone crest and cement-enamel junction, as well as show at least two posterior approximating teeth to be included. Exclusion criteria used were: 1) patients that were not within the specified age range, 2) patients with no BW radiographs, 3) patients with radiographs in which the cement-enamel junction (CEJ) and alveolar bone crest were not visible, 4) patients who did not have at least 2 approximating teeth or where the interproximal space was too narrow to observe the bone crest. Teeth were excluded if dental restorations obliterated the CEJ, rendering the distance between CEJ and alveolar crest questionable. Additionally, cases in which a tooth was found adjacent to an edentulous site with alveolar bone levels greater than 2 mm from the CEJ were not considered pathological due to possible surgical trauma. Any records indicating sites receiving osseous surgery or bone grafts were excluded. Patients were excluded as well due to closed electronic files or because their BW radiographs could not be calibrated with the measuring tool. Third molar teeth were not included due to their tendency of not being captured by BW radiographs. Non-functional teeth were excluded for the possibility of super eruption. For longitudinal data analysis, we required that eligible subjects for inclusion to have at least two exposures of complete mouth survey radiographs or repeated BW radiographs with at least one-year interval.

### Primary predictor

The main predictor was whether or not the subjects had reported taking oral BIS. Other variables included in the model were age, sex (although we did not expect any confounding by age or sex, since the two groups were matched on them, we included them to account for any residual confounding), smoking status, median house income, race, diabetes and hypertension. All data were collected from the electronic health record of the Harvard Dental Center using AxiUm® software. Due to the small number of this sample, categorization to different age categories resulted in groups with very few subjects (presented in the descriptive statistics section). Hence, age was used as a continuous predictor for the multivariable analysis. Furthermore, we categorized body mass index (BMI) into two groups of Underweight/Normal weight and Overweight/Obese, with the former group as the reference group for the same reason of scarce data.

In this sample, no one had reported as being current smoker so we created a binary smoking variable for analysis by coding everyone who had ever smoked (former smoker) as ever smoker (=1) and those who had never smoked as never smoker (=0).

### Primary outcome

The primary outcome is the mean of the alveolar bone level on mesial and distal sites of posterior teeth in millimeters between the group that was taking oral BIS and the group that was not. The bone levels at the follow-up visits were compared to the baseline mean of both groups. Interproximal bone loss occurs when the distance between the cement-enamel junction (CEJ) and the alveolar bone crest is greater than or equal to 2 mm, as determined on a bitewing radiograph [26–31]. We also classified the amount of bone loss based on the American Academy of Periodontology (AAP) case definition into mild, moderate and severe periodontitis to estimate the prevalence [26]. One trained examiner (MH) carried out the measurement of the outcome using the calibrated measuring tool of Emago® after conducting the inter-examiner reliability test. An intra class correlation coefficient (ICC) test was performed with an excellent average score of 0.96 (0.93–0.97).

### Sample size

This is a subpopulation of a large sample size (*N* = 1131) collected to estimate prevalence of periodontal diseases in HSDM. After identifying patients who reported using bisphosphonates, we implemented very strict exclusion criteria (described before). We further selected patients on the availability of repeated radiographs that affected the total number of eligible patients. We collected records of all patients that reported receiving oral BIS from 2008 to 2015 (*N* = 30), to identify suitable radiographs for analysis. We identified 26 patients out of the 30 identified earlier that satisfied inclusion criteria described before. The 26 patients who were taking BIS were then matched on age and sex to another 26 patients who did not report receiving BIS at any point of their life. Radiographs of a total of 52 patients (26 patients of each group) were analyzed over a two-year period.

It is true sample size is quite small due to the low number of patients reported taking BIS. To address this problem we calculated the power of detecting at least 0.5 mm difference between the two groups. The main sample of this subpopulation had an average mean alveolar bone level of 1.38 mm (±0.7). Given these parameters, with α set to 0.05, we have more than 80% power to detect a real difference. This could also mean that the two groups might differ in less than 0.5 mm of mean alveolar bone level. However such a difference might be considered clinically insignificant.

### Statistical analyses

Descriptive statistics of categorical data as well as the prevalence of each periodontitis case definition were calculated. A mixed-effect linear regression model with a multi-level design was performed to estimate the difference of change in mean bone level in millimeters (mm). We included the time term to adjust for the amount of change across the years of follow up for both groups.

## Results

### Descriptive statistics (univariate analysis)

A total of 52 matched subjects were included in the final analysis. Median age of participants was 70 year-old (IQR: 64–78) (Table 1). African American race was the fewest in this sample, composing almost 2%, while 54% of the sample was White. Table 2 presents different racial groups and other predictors with their measured mean bone levels. The BIS group mean alveolar bone level at baseline was 1.90 mm (±0.040) and 1.99 mm (±0.036) for the group that is not taking BIS. Of the subjects, 21% were former smokers and none of the subjects have reported themselves as current smokers.

**Table 1:** Prevalence of mild, moderate, and severe periodontitis comparing both groups of patients at baseline

	*N*	Median Age	IQR	Females %	Mild %	Moderate %	Severe %
Total	52	70	64–78	92.3	94.2	50.0	7.7
BIS							
Yes	26	70	64–78	92.3	96.1	38.4	7.7
No	26	70	64–78	92.3	92.3	61.5	7.7

**Table 2:** Descriptive statistics and prevalence of mild, moderate, and severe periodontitis of the whole sample at baseline

Percentage (%)									
	*N*	Mild	SE	Moderate	SE	Severe	SE	MABL (mm)^a^	SE
Total	52 (100)	94.2	3.2	50.0	7.0	7.7	3.7	1.94	0.027
Age Groups (yrs.)									
50–64	14 (26.9)	100.0	0.0	50.0	13.8	7.1	7.1	2.02	0.050
65+	38 (73.1)	92.1	4.4	50.0	8.2	7.9	4.4	1.91	0.032
Gender									
Male	4 (7.7)	100.0	0.0	25.0	25.0	0.0	n/a	1.76	0.067
Female	48 (92.3)	93.7	3.5	52.1	7.2	8.3	4.0	1.96	0.029
Race									
White	28 (53.9)	96.4	3.5	53.5	9.6	7.1	4.9	1.98	0.035
African American	1 (1.9)	100.0	0.0	100.0	0.0	0.0	n/a	2.45	0.232
Asian	6 (11.5)	100.0	0.0	83.3	16.6	33.4	21.1	2.19	0.106
Other	4 (7.7)	100.0	0.0	25.0	25.0	0.0	n/a	1.50	0.074
Unknown	13 (25)	88.9	11.1	33.4	16.7	0.0	n/a	1.83	0.047
Median House Income									
Low	16 (30.7)	87.5	8.5	68.7	11.9	12.5	8.5	2.08	0.051
High	36 (69.3)	97.2	2.7	41.7	8.3	5.5	3.8	1.88	0.031
Body Mass Index									
Underweight	2 (3.8)	100.0	0.0	100.0	0.0	0.0	n/a	2.29	0.119
Normal	18 (34.6)	100.0	0.0	50.0	12.1	5.6	5.6	1.91	0.043
Overweight	10 (19.2)	80.0	13.3	30.0	15.2	10.0	10.0	1.68	0.064
Obese	4 (7.7)	75.0	25.0	25.0	25.0	0.0	n/a	1.57	0.070
Not reported	18 (34.6)	100.0	0.0	61.1	11.8	11.1	7.6	2.16	0.048
Smoking Status									
Never smoker	13 (25)	84.6	10.4	46.1	14.4	7.7	7.7	1.73	0.047
Former smoker	11 (21.1)	100.0	0.0	45.5	15.7	18.2	12.2	2.05	0.064
Current Smoker	0	0.0	n/a	0.0	n/a	0.0	n/a	0.0	n/a
Not reported	28 (53.9)	96.4	3.5	53.5	9.6	3.5	3.5	2.0	0.037
Bisphosphonate intake									
Yes	26 (50)	96.1	3.8	38.4	9.7	7.7	5.3	1.90	0.040
No	26 (50)	92.3	5.3	61.5	9.7	7.7	5.3	1.99	0.036
Diabetes									
Yes	2 (3.9)	100.0	0.0	0.0	n/a	0.0	n/a	1.54	0.102
No	50 (96.1)	94.0	3.4	52.0	7.1	8.0	3.8	1.95	0.027
CVD									
Yes	15 (28.9)	93.4	6.6	40.0	13.1	6.7	6.7	1.91	0.048
No	37 (71.1)	94.6	3.7	54.0	8.3	8.1	4.5	1.95	0.032
Hypertension									
Yes	35 (67.3)	94.1	5.8	35.3	11.9	11.7	8.0	1.85	0.047
No	17 (32.7)	94.2	3.9	57.1	8.4	5.7	3.9	1.98	0.033

^a^Mean alveolar bone level in millimeters

### Severity of the disease based on case definitions

**Fig. 1:**
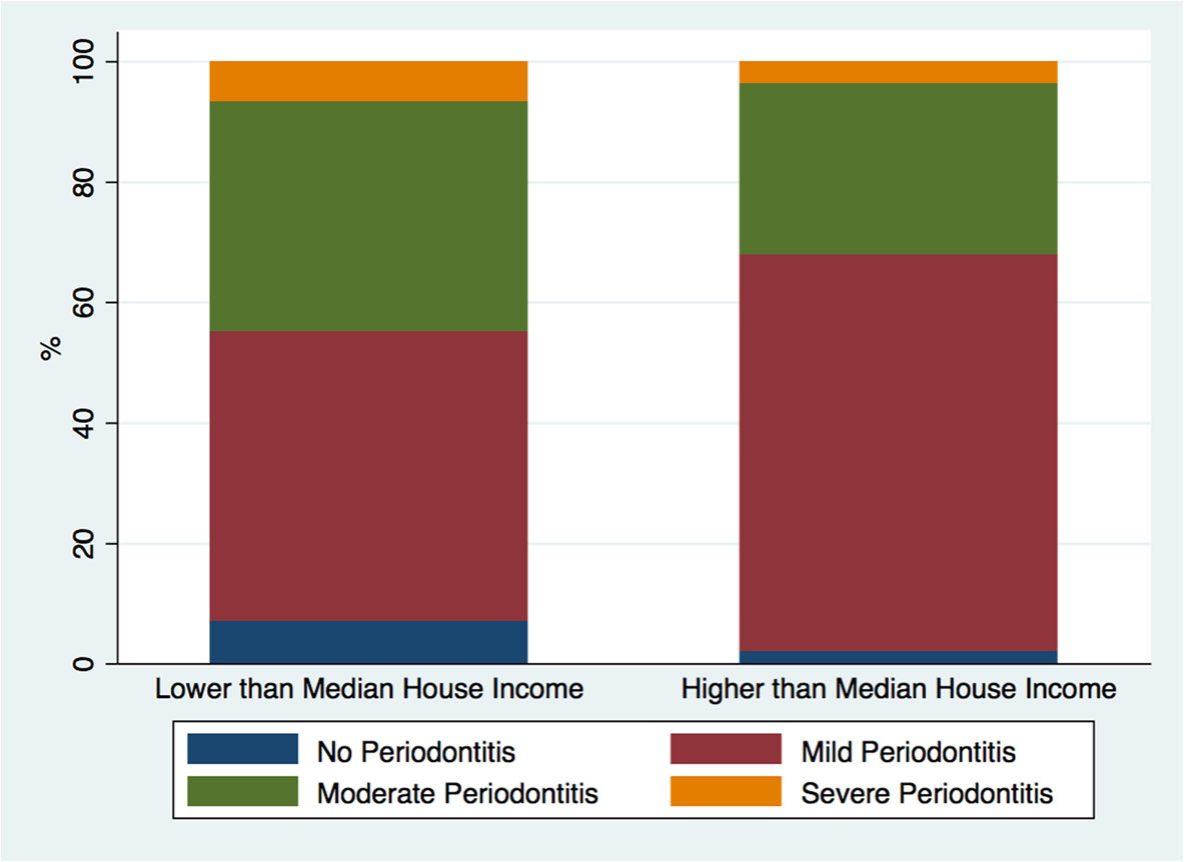
Prevalence of mild, moderate, and severe periodontitis by median house income

The overall prevalence of mild periodontitis was 94.2% (±3.2), moderate periodontitis was 50% (±7.0) and severe periodontitis was 7.7% (±3.7) (Table 2). Mild periodontitis was higher in the BIS group compared to the no BIS group; however, moderate periodontitis was higher in the no BIS group (Table 1). Moreover, moderate and severe periodontitis were higher among individuals with low median house income (Fig. 1).

### Unadjusted estimates overtime (bivariate analysis)

After the two-year interval, the group with no history of receiving oral BIS did not experience significant change in mean bone level. On the other hand, the BIS group had experienced 0.087 mm mean bone loss after 2 years with marginally statistical significance compared to the group with the no BIS intake baseline (95% CI: − 0.0002, 0.175. *P*-value = 0.051). Table 3 presents the bivariate analysis and its unadjusted estimates of mean bone loss at baseline and over time.

**Table 3:** Crude and adjusted mean alveolar bone loss (mm) for both groups over time

Variables	Adjusted MABL (mm)^a^ 95% CI	*p*-value	Unadjusted MABL (mm)^a^ 95% CI	*p*-value
Year^a^BIS				
0 No BIS (reference)				
2 No BIS	−0.027(−0.08,0.03)	0.374	−0.027(−0.08,0.03)	0.383
0 BIS+	0.084(−0.16,0.033)	0.515	−0.059(− 0.27,0.15)	0.594
2 BIS+	0.088 (0.001,0.176)	0.048	0.087(−0.0002,0.175)	0.051
Age (continious yrs)				
1 year increase	− 0.002(− 0.016,0.012)	0.764		
Gender				
Female (reference)				
Male	−0.312(− 0.830,0.204)	0.236		
Race				
White (reference)				
African American	0.476(−0.244,1.198)	0.195		
Asian	0.092(−0.246,0.432)	0.591		
Other	−0.289(− 0.708,0.129)	0.176		
Unknown	−0.108(− 0.348,0.130)	0.373		
Median House Income^a^				
Low (reference)				
High	−0.153(−0.405,0.098)	0.233		
Body Mass Index				
Underweight/Normal (reference)				
Overweight/Obese	−0.235(−0.476,0.004)	0.055		
Smoking Status				
Never smoker (reference)				
Former smoker	0.153(−0.199,0.505)	0.394		
Current Smoker	n/a	n/a		
CVD				
No (reference)				
Yes	0.133(−0.165,0.433)	0.381		
Hypertension				
No (reference)				
Yes	−0.118(−0.388,0.150)	0.388		
D4341				
No (reference)				
Yes	0.113(−0.169,0.396)	0.433		
Random effect				
Between Individuals	0.14 (0.10,0.17)	n/a		
Between Teeth	0.12 (0.10,0.13)	n/a		
Between Sites	0.21 (0.19,0.22)	n/a		

*N* = 52 patients (2307 sites from 658 teeth)

^a^Mean alveolar bone loss in millimeter

### Adjusted estimates over time (multi-variable analysis)

Since subjects were matched on age and sex, we did not expect adding these two variables to the model would affect the outcome significantly. However, we included them to control for any residual confounding by age or sex. None of the variables included in the model showed significant association with the outcome. For the group who did not take oral BIS, change over time was not significant after the two-year period. However, the BIS group had experienced 0.088 mm more bone loss compared to the no BIS group (95% CI: 0.001, 0.176. *P*-value = 0.048), adjusting for all other variables included in the model. Table 3 presents the estimates at baseline and over time, in addition to the estimates of all other variables. A possible explanation of this observation is that the no BIS group received double the number of periodontal treatments (scaling and root planing) in the measured teeth after the first included x-ray, compared to the BIS group, using Current Dental Terminology (CDT) codes from American Dental Association (Table 4).

**Table 4:** Proportion of patients received periodontal procedures including scaling and root planing comparing BIS and no BIS groups

		N()	N(%)
CDT Code		BIS = 1	BIS = 0
D4261	Osseous surgery for one to three teeth	2 (7.7)	0 (0)
D4263	Bone replacement graft	3 (11.5)	4 (15.4)
D4265	Biologic materials – tissue regeneration	3 (11.5)	3 (11.5)
D4266	Guided tissue regeneration	1 (3.8)	0 (0)
D4341	Scaling/root planing for 4 teeth or more	2 (7.7)	6 (23)
D4342	Scaling/root planing for 1–3 teeth	6 (23)	10 (38.4)
None		9 (34.6)	3 (11.5)
Total		26 (100)	26 (100)

*CDT* Current Dental Terminology

*N* = 52 patients

### Random-effect estimates

The estimates (mean change) of random effect vary between individuals and teeth. It was 0.14 mm (95% CI: 0.10, 0.17) and 0.12 mm (95% CI: 0.10, 0.13), respectively. Random-effect coefficients are also provided in Table 3.

## Discussion

Results of this study indicate that, after 2 years of follow up, the oral administration of BIS did not have a protective effect on the mean alveolar bone loss. Although a recent systematic review and meta-analysis on the effect of BIS used as an adjunctive treatment of periodontal diseases indicated the beneficial effect of BIS administration, the authors concluded that due to short periods of follow up in the eight studies identified in the literature, as well as the potential adverse effect of BIS in the oral cavity–osteonecrosis of the jaws, its use as an adjunctive treatment for managing periodontal diseases is debatable [23].

Another study, that was not included in the previously mentioned systematic review, was published by Jeffcoat et al. in 2007 to investigate the effectiveness of oral alendronate [17]. In that study, 335 patients were randomized into two groups of alendronate and no-drug groups and were followed over 24 months. After 2 years of follow up, the group receiving oral alendronate did not show any significant change in either alveolar bone density or alveolar bone loss compared to the control group.

Only patients that were having low mandibular bone mineral density at baseline showed significant reduction of bone loss compared to the control group. The authors of the study concluded that administering oral alendronate over 2 years for patients with periodontitis had no effect on alveolar bone loss except for the subpopulation of patients who had low mandibular bone mineral density.

Although studies that examined the effect of oral BIS disagreed on its effect on periodontal health [16, 17, 22, 24], the route of administration may play an integral role in the effectiveness of bisphosphonate on alveolar bone loss.

The local delivery of 1% alendronate gel was also examined in patients with aggressive periodontitis (a more severe form of periodontal disease [32], and diabetic patients with chronic periodontitis (a systemic disease that is associated with a higher risk of developing periodontal diseases [33], as an adjunct to scaling and root planing for the treatment of intrabony defects. The researchers of both studies found a significant reduction in probing depth, greater gain of clinical attachment level, and bone reforming of intrabony defects. Moreover, an animal study conducted by Price et al., found that the local delivery of a simvastatin-alendronate-β-cyclodextrin was statistically associated with reduced bone loss as a consequence of periodontitis [34].

A partial-mouth periodontal examination would result in underestimating the true change in mean bone loss. However, we did not have missing outcomes related to loss to follow up (lack of radiographs); all 52 patients were followed for 2 years. Nevertheless, the sample size was relatively small, having only 26 patients in each group which may not representative of the entire population. However, the results of this research did not contradict our current knowledge using longitudinal analysis. Moreover, the BIS group may have exhibited underlying factors that affected their bone biology and resulted in an increased risk of bone loss that was not observed on this small group of patients, such as purpose for receiving it (part of the treatment for osteoporosis, malignant condition or systemic steroids), duration of use and doses.

## Conclusion

Bisphosphonate medications are indicated for several bone-related diseases. In our study, we found that the group who reported receiving oral bisphosphonates showed no improvement in maintaining alveolar bone level—on the contrary, our results suggest that the use of oral BIS may not be effective in reducing annual alveolar bone loss. However, further investigation may be needed to investigate its role as an adjunct in periodontal therapy and the effect of treatment modalities on bone response. The implication of this study, however, may indicate that the route of administration of bisphosphonate plays an important role for its effectiveness to be achieved. Emerging evidence of several studies indicates that the local delivery of bisphosphonate can help in maintaining periodontal health and alveolar bone level for patients who are more prone to the periodontitis.

## Data Availability

The dataset used during the study are available from the corresponding author upon request.

## Abbreviations

AAP: American Academy of Periodontology
BIS: Oral bisphosphonate
BW: Bitewing
CEJ: Cement-enamel junction
CMRS: Complete Mouth Radiographic Series
DBP: Diastolic blood pressure
SBP: Systolic blood pressure
